# Magnetic resonance angiography derived predictors of progressive dilatation and surgery of the aortic root in Marfan syndrome

**DOI:** 10.1371/journal.pone.0262826

**Published:** 2022-02-03

**Authors:** Julius Matthias Weinrich, Alexander Lenz, Gerhard Schön, Cyrus Behzadi, Isabel Molwitz, Frank Oliver Henes, Bjoern Philip Schoennagel, Gerhard Adam, Yskert von Kodolitsch, Peter Bannas

**Affiliations:** 1 Department of Diagnostic and Interventional Radiology and Nuclear Medicine, University Medical Center Hamburg-Eppendorf, Hamburg, Germany; 2 Department of Medical Biometry and Epidemiology, University Hospital Hamburg-Eppendorf, Hamburg, Germany; 3 Department of Cardiology, University Heart Center Hamburg, University Hospital Hamburg-Eppendorf, Hamburg, Germany; Nagoya University, JAPAN

## Abstract

**Background:**

To identify magnetic resonance (MR) angiography derived predictors of progressive dilatation and surgery of the aortic root in Marfan syndrome.

**Material and methods:**

We retrospectively included 111 patients (32.7±16.5 years, range: 7–75 years) with a total of 446 MR angiographies. Aortic diameter growth rates of the entire thoracic aorta and Z-scores were estimated from annual diameter measurements. Aortic root shape was subdivided into three different types: (T0) normal; (T1) localized dilatation; (T2) generalized aortic root dilatation. Aortic diameter, Z-score, age, and aortic root shape at baseline were tested as predictors of aortic root dilatation using a multivariate logistic regression model.

**Results:**

The highest aortic growth rate was observed at the level of the sinuses of Valsalva. Higher aortic root diameters and Z-scores at baseline predicted an increased growth of the aortic root (p = 0.003 and p<0.001). Young age (<30 years) was a predictor for the increase of Z-scores when compared to patients ≥30 years (p = 0.019). 25/111 patients (22.5%) had a T0 aortic root shape, 59/111 patients (53.2%) had a T1 aortic root shape, and 27/111 patients (24.3%) had a T2 aortic root shape. Aortic root shape did not predict further aortic growth (p>0.05). However, significantly more patients undergoing surgery had a generalized aortic dilatation (19/28, 76.9%) than a localized aortic root dilatation (9/28, 32.1%) (p = 0.001).

**Conclusion:**

Larger baseline aortic root diameter and Z-score as well as young age predict solely progressive aortic root dilatation in Marfan patients. MR angiography derived type of aortic root shape does not predict aortic growth, but patients with generalized aortic root dilatation are referred more frequently for aortic surgery.

## Background

Marfan syndrome is a hereditary connective tissue disorder caused by mutations in the *FBN1* gene encoding the protein fibrilin-1 [1]. Progressive aortic root dilatation is the most frequent cardiovascular complication in Marfan patients [1, 2]. Aortic root aneurysms may lead to aortic dissection and represent the main cause for a decreased life expectancy in Marfan patients [1–4].

Current guidelines recommend surgical repair of the dilated aortic root/ascending aorta at a threshold-diameter of 50 mm or even less in Marfan patients with additional risk factors, i.e. rapid growth defined as an increase of aortic dimensions greater than 0.5 cm/y [5, 6].

However, aortic growth rates among Marfan patients vary widely [7] and factors predicting the rate of aortic growth are poorly understood [8, 9]. The risk of aortic dissection in Marfan patients increases not only with aortic root diameters but is also associated with an increased growth rate. Therefore, it is of clinical importance to identify possible predictors of rapid enlargement of the aortic root diameter [10–12]. Echocardiography derived features such as larger baseline aortic root dimensions or generalized aortic root dilatation have been associated with progressive aortic root enlargement [7, 13–15].

However, echocardiography cannot assess the entire aorta and is highly operator-dependent [16]. Magnetic resonance (MR) angiography allows to cover the entire aorta with excellent image quality [17–19]. Hence, MR angiography allows for accurate visualization of the aortic root morphology and exact diameter measurements of the entire thoracic aorta in Marfan patients [17, 20]. We hypothesized, that the aforementioned advantages of MR angiography may improve the predictive power of different aortic root diameters and shapes for progressive aortic root dilatation in Marfan patients [21]. Therefore, the aim of this study was to derive from MR angiography improved predictors of progressive dilatation and surgery of the aortic root in Marfan syndrome.

## Material and methods

The local institutional review board (Ärztekammer Hamburg, Germany) approved our retrospective single-center study and waived the requirement for informed consent as all data was analyzed anonymously.

### Study population

We identified retrospectively 160 consecutive Marfan patients (64 males, 96 females; age range 7–75 years; mean 32.7±16.5 years) without prior aortic surgery from our database who underwent annual MR angiography between September 2005 and July 2017.

All patient records were searched for date of aortic complications and/or aortic surgery. Marfan diagnosis was established according to the latest Ghent nosology and all patients underwent genetic analyses with sequencing of the *FBN1* gene. *FBN1* gene changes fulfilled ≥1 Ghent criteria of causality as defined by the current Ghent nosology in all patients and a pathologic variant was identified in all patients [22, 23].

### MR angiography

MR angiography of the entire aorta was performed with or without [18, 20] contrast enhancement as described previously using either 1.5 [24] or 3 Tesla [25] MR systems equipped with multi-channel receiver coils for cardiovascular imaging (Achieva and Ingenia, Philips Medical Systems, Best, The Netherlands). All images were interpreted on state-of-the-art RIS/PACS workstations (Centricity^™^ RIS-I 4.2 Plus, GE General Electric Company).

### Image analyses

**Fig 1:**
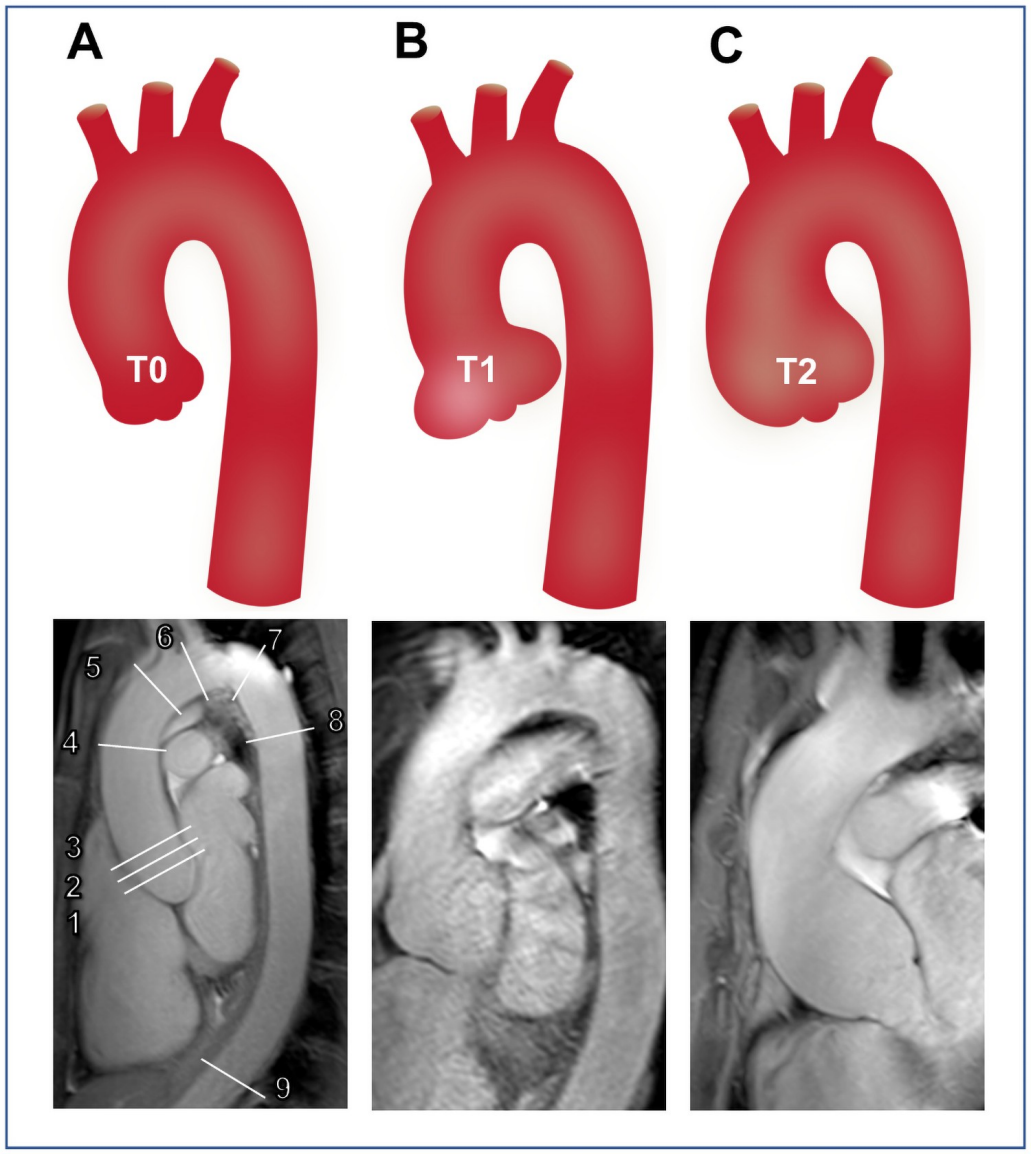
Illustration of different aortic root shapes (upper panels) in Marfan patients and corresponding MR angiography (lower panels). **(A)** normal shape of the aortic root without dilatation (T0). **(B)** Localized aortic root dilatation, which is confined to the sinuses of Valsalva (T1). **(C)** Generalized aortic root dilatation with extension beyond the sinotubular junction (T2). MR angiography in para-sagittal orientation of Marfan patients with **(A)** normal shape of the aortic root without dilatation (T0), **(B)** Localized aortic root dilatation (T1), and **(C)** generalized aortic root dilatation (T2). Diameter measurements were performed at the level of the aortic annulus (1), sinuses of Valsalva (2), sinotubular junction (3), mid ascending aorta at the pulmonary artery bifurcation (4), at the innominate artery (5), aortic arch between the left common carotid and left subclavian artery (6), proximal descending aorta at isthmus, 2 cm distal to left subclavian artery (7), mid descending aorta (at pulmonary artery bifurcation) (8), and thoraco-abdominal aorta at the level of the diaphragm (9). Of note, readers were presented the entire para-sagittal stack of images and were free to choose appropriate slices displaying the maximal profile of the aorta.

The shape of the aortic root was categorized into three types based on the morphology in coronal and para-sagittal imaging planes. Patients without any changes of aortic root shape were classified as normal without dilatation (type 0 = T0). The shape of aortic root dilatation was termed as localized aortic root dilatation (type 1 = T1) when confined to the sinuses of Valsalva and as generalized aortic root dilatation (type 2 = T2) if enlargement extended beyond the sinotubular junction (Fig 1) [26].

Diameter measurements were performed at nine levels of the thoracic aorta: aortic annulus (1), sinuses of Valsalva (2), sinotubular junction (3), mid ascending aorta (4), at the innominate artery (5), aortic arch between the left common carotid and left subclavian artery (6), proximal descending aorta at isthmus, 2 cm distal to left subclavian artery (7), mid descending aorta (8), and thoraco-abdominal aorta at the level of the diaphragm (9) (Fig 1) [17, 27]. Diameter measurements were performed perpendicular to the blood-filled lumen at all levels on identically orientated para-sagittal MR angiography images and readers were free to choose slices displaying the maximal aortic diameter. Using identically oriented para-sagittal images avoided possible user influence introduced by individually performed multiplanar reformations [20].

### Calculation of Z-scores

Z-scores for each Marfan patient were calculated based on the absolute aortic diameter at the level of the sinuses of Valsalva, age, and body surface area. Z-scores for aortic diameters express the deviation from a normative size- or age-specific population mean. A Z-score between −2 and +2 is considered normal [28]. For children ≤ 18 years the equation was based on MR angiography derived normative values provided by Kaiser et al. [29]. There is currently no MR angiography specific normative data for adult patients. Therefore, we used echocardiographic derived data provided by Devereux et al. [30] to calculate Z-scores at the sinuses of Valsalva for patients > 18 years. Patients who were initially aged <18 years and crossed into the 18–30 years group over the course of the study period were analyzed solely with normative values provided by Kaiser et al. or Deveraux et al. Patients were categorized to either one method based on the age group in which the majority of measurements was performed.

### Statistical analyses

Growth rates for all aortic levels were calculated in all patients with ≥2 MR examinations by applying a locally estimated scatterplot smoothing (LOESS) to describe changes in diameter.

**Fig 2:**
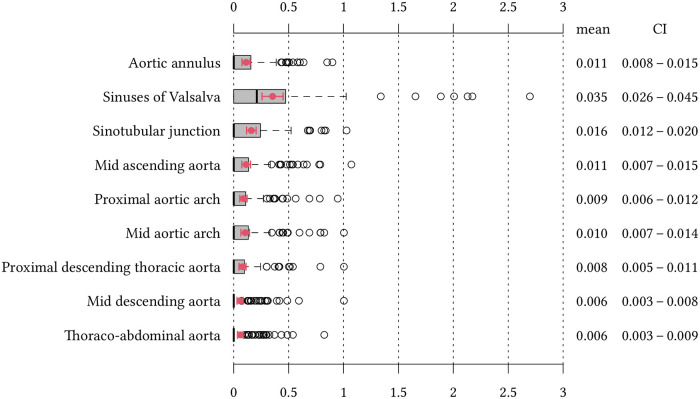
Boxplot analyses of naive growth rates at different aortic levels in 111 Marfan patients. Median naive (i.e., not adjusted) growth rates are indicated for each aortic level by thick vertical lines. The highest median growth rate was observed at the level of the sinuses of Valsalva. The right end of the box indicates the upper quartile and whiskers indicate the highest value within 1.5 IQR. Arithmetic naive mean and confidence intervals are displayed in red. Circles indicate outliers above the whiskers.

To determine the importance of diameter at first measurement, age, and the pattern of aortic root on aortic growth, we calculated a multivariate regression model, a slopes as outcome model. The dependent variable was the annual growth in mm, and the independent variables were aortic diameter at the time of first measurement, three different age groups (<18 years, 18–30 years and ≥ 30 years), sex, treatment with antihypertensive medication and the pattern of aortic root. Hence, annual growth was adjusted for the independent variables. Results were reported as regression coefficients, confidence intervals of the coefficients and p-values as well as estimated marginal means with their corresponding 95% confidence intervals (95%-CI), which are presented in graphs. Also, we report naive aortic measurement values in Fig 2 which are consequently not adjusted for the above-mentioned independent variables. Frequency of elective surgery between aortic root shape (T1, T2) was compared using the Pearson chi-squared test.

Data were collected and analyzed with an Excel spreadsheet (v. 15.0 Microsoft, Redmond, WA, USA). All statistical analyses were computed using R version 4.0.4 (R Core Team, Vienna, Austria, 2021). P values <0.05 indicated statistically significant differences.

## Results

A total of 495 MR angiographies was performed in the included 160 Marfan patients. All MR angiography examinations were performed with diagnostic image quality.

### Growth rates at different aortic levels

Of the 160 patients, 111 patients (69.4%) (43 males, 68 females age range 9-74 years, mean age 33.2±16.4 years) underwent ≥ 2 consecutive MR examinations (total of 446 MR angiographies) and allowed for calculation of growth rates. Patients underwent a median of 4 scans (range 2–11). The time interval between individual scans was 12 ± 1 months as all patients were scanned as part of their yearly workup in our Centre of Expertise for Marfan syndrome. Accordingly, the median monitoring time was 4.5 years (interquartile range: 4.2; range 2–11 years) (Table 1).

**Table 1:** Demographics and MR-angiography data.

	n	Mean age (standard deviation)
All patients	111	33.1 ± 16.4
Age group <18 years	37	12.5 ± 3.2
Age group 18–30	27	23.2 ± 3.6
Age group ≥ 30 years	47	44.2 ± 11.5
Female	68	
MR-Angiographies		
Total (n)		446
Median number of MRA/patient (n)		4.0
Median monitoring time (years)		4.5
Time interval between scans (months)		12 ± 1

There were 37 patients in the age group <18 years (mean age 12.5±3.2 years), 27 patients in the age group 18–30 years (mean age 23.2±3.6 years), and 47 patients in the age group ≥ 30 years (mean age 44.2±11.5 years) (Table 1).

The highest naive aortic growth rates were observed at the level of the sinuses of Valsalva (0.035 mm/year (95%-CI: 0.026–0.045) and the lowest naive growth rates were observed in the mid descending aorta (0.006 mm/year (95%-CI: 0.003–0.008). Fig 2 demonstrates naive growth rates at all nine levels of the thoracic aorta.

The remaining 49/160 patients (30.6%) (21 males, 28 females; age range 7-75 years; mean 36.8±16.1 years) underwent only one MR angiography, precluding calculation of growth rates.

### Predictors of aortic root diameter growth

**Fig 3:**
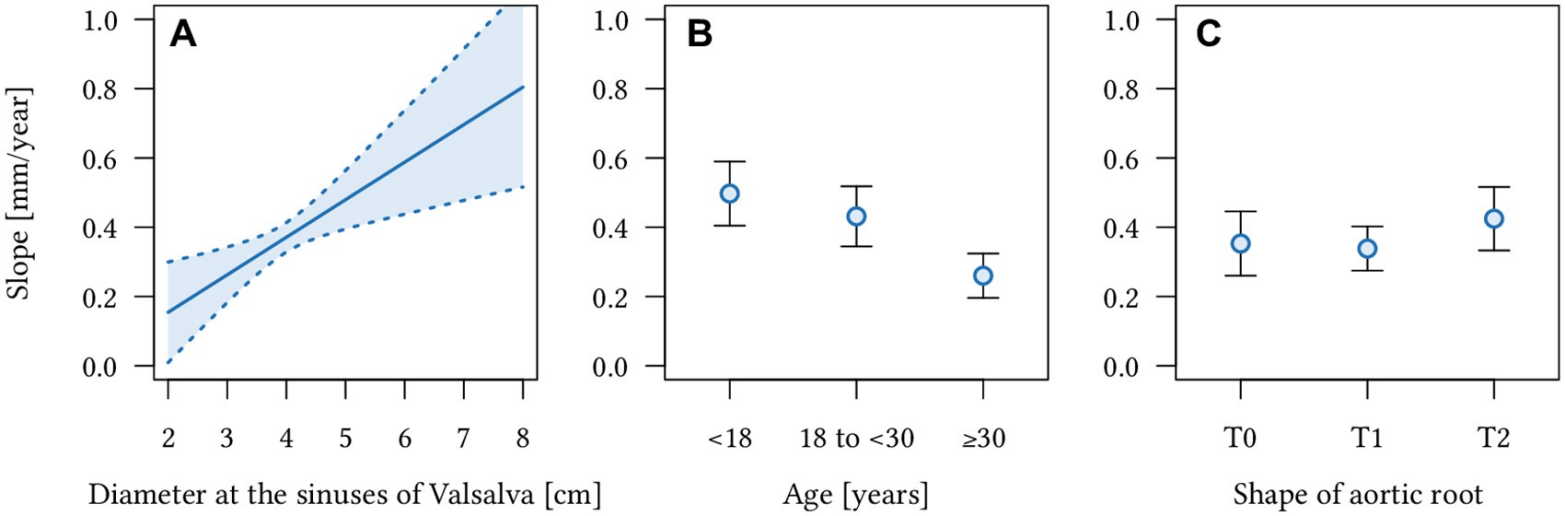
Effect plots with 95% confidence intervals for adjusted growth rates of absolute aortic diameters at the level of the sinuses of Valsalva in 111 Marfan patients. Results of multivariate regression with **(A)** diameter at baseline, **(B)** age and **(C)** shape of aortic root as dependent variables and adjusted growth rate of aortic diameters as outcome variable. Larger aortic root diameter at baseline **(A)** and younger age **(B)** are significantly associated with higher adjusted growth rates of the aortic root at the level of the sinuses of Valsalva. **(C)** There is no significant association between the shape of the aortic root (T0, T1, or T2) and the adjusted aortic growth rates.

The mean aortic root diameter at baseline was 3.3±0.6 cm (Z-score: 2.8±2.1) in the age group <18 years, 3.9±0.5 cm (Z-score: 3.3±1.7) in the age group 18–30 years and 4.0±0.5 cm (Z 2.8±1.7) in patients ≥ 30 years. Multivariate regression analyses revealed that larger absolute aortic root diameters at baseline predicts the increase of absolute aortic root diameters and Z-scores (p = 0.003 and p<0.001) (Table 2). Aortic root diameters at baseline are associated with growth rates of absolute aortic diameters at the sinuses of Valsalva (Fig 3A). Based on the multivariate regression model the estimated adjusted growth rate at the sinuses of Valsalva increases from 0.26 mm/year in patients with a baseline diameter of 30 mm (95%-CI: 0.18-0.34 mm/year) to 0.5 mm/year (95%-CI: 0.52–1.09 mm/year) in patients with aortic root baseline diameters of 50 mm (Fig 3A).

**Table 2:** Results of multivariate analyses predicting growth rate at the sinuses of Valsalva (SOV).

	p-value	
	Z-score	Diameter
Baseline diameter/Z-score SOV	<0.001	0.003
age group ≥ 30 years	0.019	<0.001
age group 18–30 years	n.s	n.s
Aortic root shape T1	n.s	n.s
Aortic root shape T2	n.s	n.s
Antihypertensive medication	n.s	n.s
Sex: male	n.s	n.s

The mean age at baseline was 33.2±16.4 years and ranged from 9 to 74 years in the included 111 patients. Multivariate regression analyses revealed that young age (<30 years) predicts for increased adjusted diameter growth of absolute aortic root diameter (p<0.001) and Z-score (p = 0.019) when compared to patients ≥30 years (0.26 mm/year, 95%-CI: 0.19–0.32). There was no significant difference between adjusted growth rates of absolute aortic root diameters (p = 0.326) and z-scores (p = 0.460) in patients <18 years (0.5 mm/year, 95%-CI: 0.40–0.59) when compared to patients 18-<30 years (0.43 mm/year, 95%-CI: 0.34-0.52) (Fig 3B).

Aortic root shape was normal (T0) in 25 of the 111 patients (22.5%). Localized aortic root dilatation (T1) was present in 59 patients (53.2%) and 27 patients (24.3%) had a generalized aortic root dilatation (T2). Multivariate regression analyses revealed that neither localized (T1) nor generalized (T2) aortic root dilatation allow prediction of diameter growth of the aortic root for absolute diameters (both p>0.32) or Z-scores (both p>0.26).

Adjusted growth grates of the aortic root were 0.35 mm/year (95%-CI: 0.26–0.44) for patients with normal aortic root shape (T0), 0.34 mm/year (95%-CI: 0.27–0.40) for patients with localized root dilatation (T1), and 0.42 mm/year (95%-CI: 0.33–0.52) for patients with generalized aortic root dilatation (T2) (Fig 3C).

**Fig 4:**
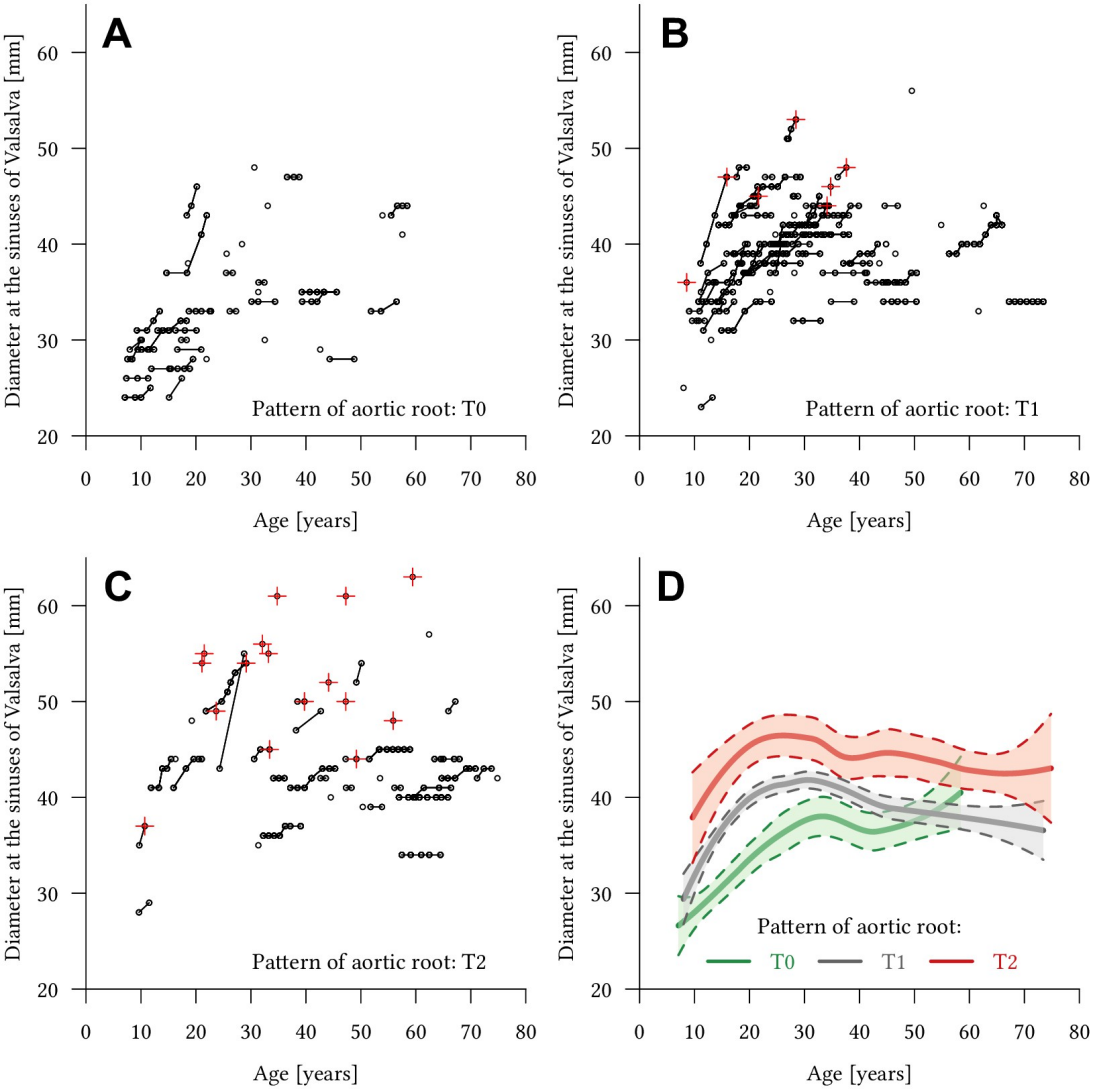
Spaghetti graphs of absolute aortic root diameters for different shapes of the aortic root in Marfan patients. Spaghetti graphs for all 160 Marfan patients with **(A)** normal aortic root shape (T0), **(B)** localized annulo-aortic dilatation (T1), and **(C)** generalized aortic root dilatation (T2). Red crosses indicate that the aortic MR angiography-derived aortic measurement was directly followed by aortic root replacement. Note that all of patients undergoing surgery had an altered shape of the aortic root (T1 or T2) and the high proportion of patients with generalized aortic root dilatation undergoing surgery (T2). **(D)** Local regression analysis of all three types of aortic root shape (T0 = green, T1 = grey, T2 = red) with 95% confidence intervals in all 160 Marfan patients.

**Fig 5:**
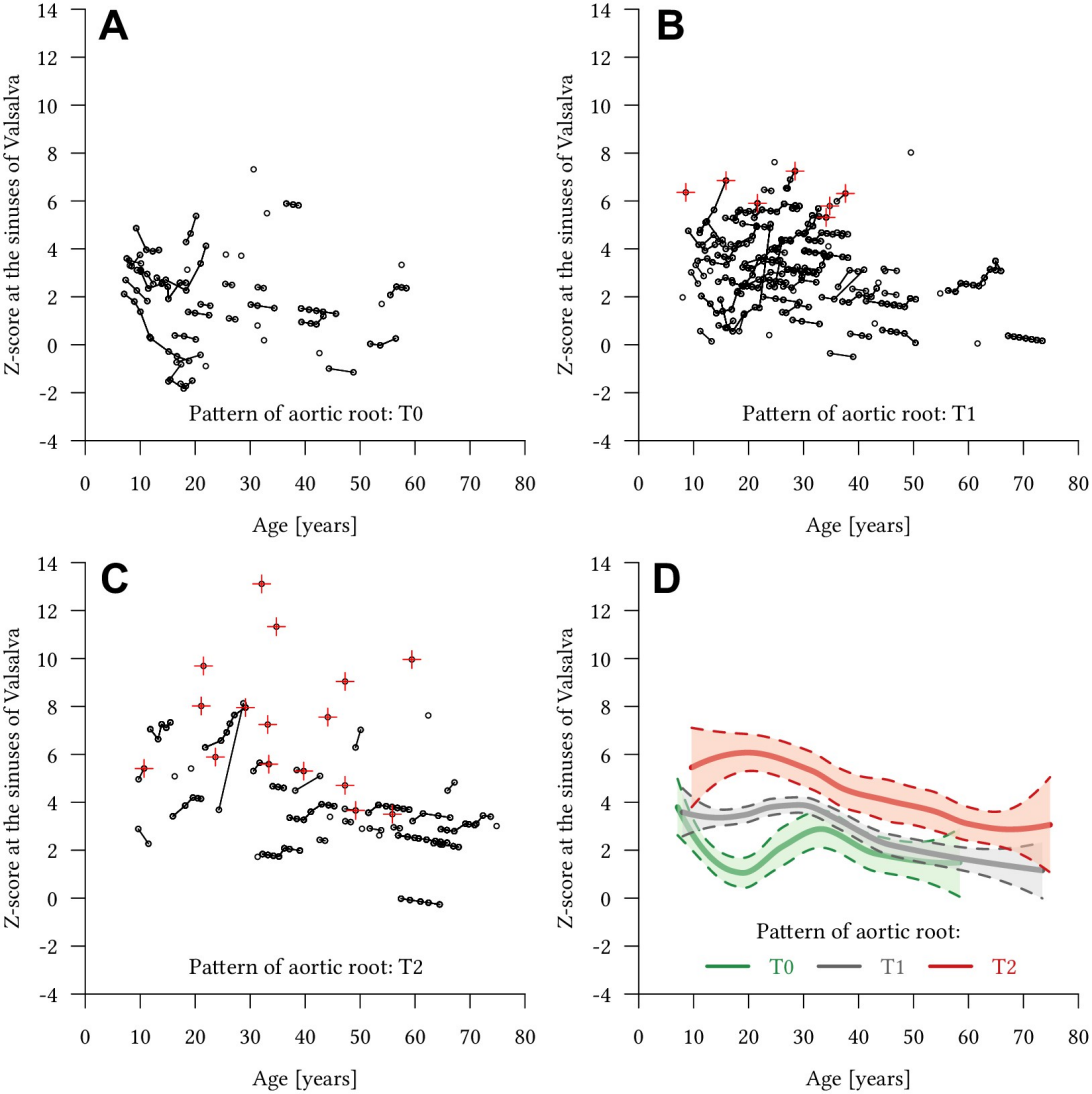
Spaghetti graphs of Z-scores for different shapes of the aortic root in Marfan patients. Spaghetti graphs for all 160 Marfan patients with **(A)** normal aortic root shape (T0), **(B)** localized annulo-aortic dilatation (T1), and **(C)** generalized aortic root dilatation (T2). Red crosses indicate that aortic MR angiography-derived Z-scores were directly followed by aortic root replacement. Note that all patients undergoing surgery had an altered shape of the aortic root (T1 or T2) and the high proportion of patients with generalized aortic root dilatation undergoing surgery (T2). **(D)** Local regression analysis of all three types of aortic root shape (T0 = green, T1 = grey, T2 = red) with 95% confidence intervals in all 160 Marfan patients.

The relation of age and aortic root shape to growth rates is illustrated as Spaghetti graph models for absolute aortic diameters (Fig 4) and Z-scores (Fig 5). Both graphs emphasize the results of the multivariate regression analyses in demonstrating that aortic root growth rates are higher in patients <30 years.

Sex (p = 0.9) and treatment with antihypertensive medication (p = 0.7) did not influence growth rates of the aortic root.

During the study period, 28 of 160 Marfan patients (17.5%) were referred for aortic root surgery, these patients are indicated by red crossed in Figs 4 and 5. All of these 28 Marfan patients undergoing surgery had an altered shape of the aortic root (T1 or T2). Significantly more patients undergoing surgery had a generalized aortic dilatation (T2) (19/28, 67.9%) than a localized aortic root dilatation (T1) (9/28, 32.1%) (p = 0.001).

## Discussion

Our retrospective longitudinal study assessed MR angiography derived predictors for aortic root growth in patients with Marfan syndrome. Our results reveal that larger absolute diameters and Z-scores at baseline examination predict progressive dilatation of the aortic root. Aortic root shapes were not associated with rapid aortic growth but generalized aortic root dilatation was associated with aortic root surgery.

We demonstrated that MR angiography-based assessment of the aortic root shape does not allow reliable prediction of growth of the aortic root despite the fact that patients with generalized aortic root dilatation are referred significantly more frequent for aortic surgery.

Larger aortic root diameters at baseline were associated with progressive aortic root dilatation. This MRI derived finding is in accordance with previous echocardiographic studies demonstrating larger baseline diameters to predict progressive aortic root dilatation in Marfan patients [14, 31]. In contrast to previous echocardiographic studies, our MR angiography data allowed for diameter measurements not just of the aortic root but for the first time the entire thoracic aorta. However, our study in Marfan patients prior to aortic surgery revealed only low growth rates in the aortic arch and descending aorta. We believe that the distal aorta may be less of a concern in pre-operative Marfan patients, but it is known that Marfan patients suffer from post-surgical complications of the distal non-operated aorta after aortic root replacement [32, 33].

Young age was associated with a more progressive growth of the aortic root in our MRI-based study. This is in accordance with a echocardiographic study by Aburawi et al. who showed that the risk for aortic root dilatation is highest below the age of 19 years, emphasizing its relation to the somatic growth phase [34]. Another echocardiographic study by van Karnebeek et al. supports this finding and demonstrates a prevalence of aortic root dilatation in 43/52 patients with Marfan syndrome under the age of 16 years [35]. In contrast to these findings, both absolute diameter and Z-score growth rates were comparable for patients <18 vs. patients 18-<30 years in our study population.

In our study, growth rates decreased significantly in older patients (≥30 years). However, Kornbluth et al. demonstrated that aortic root dilatation may occur at all ages [36]. This is in line with findings by Aburawi et al. who showed that about one third of patients develop new aortic root dilatation at a higher age [34]. Hence, our results support that the risk for aortic root dilatation is at its highest during the growth phase but cannot be fully excluded in patients >30 years of age. Especially in older patients (>50 years) other factors than Marfan syndrome alone may affect the aortic growth. However, the low numbers of patients >50 years as well as lack of a matched control group preclude a meaningful subanalysis of pre-existing risk factors for aortic growth. In order to overcome this limitation an even larger number as well as a control group should be included in future studies.

Aortic root shape was not predictive of progressive aortic root dilatation in our MR angiography-based study. This observation contradicts an early echocardiographic study by Roman et al. showing that the aortic growth rate was higher for patients with generalized aortic root dilatation when compared to localized aortic root dilatation [7].

The difference between our results and previously reported echocardiographic [7] data may have different reasons. First, aortic root dilatation in Marfan patients is often asymmetric [21]. This asymmetry may not be detected by echocardiography alone whereas MR angiography overcomes limitations such as a poor acoustic window and enables superior and detailed visualization of anatomic details in three dimensions [21]. Thus, we believe that there might be a modality-based bias in the assessment of localized vs. generalized dilatation in asymmetric aortic root dilatation. However, it needs to be taken into account that our predefined levels of measurements differ from clinical practice in which the maximum dimension at any site of the aorta is taken and preset levels may not be reflective of the maximal dilatation. In this study setting we decided to measure only at defined and preset sites for a standardized characterization of aortic diameters that can be compared with results of future studies or other patient groups.

Second, estimation of growth rates was limited in our study by the fact that patients with generalized aortic dilatation frequently underwent surgery after their first MR examination, precluding calculation of growth rates in this subgroup. Also, the remaining patients with generalized aortic dilatation demonstrated relatively low growth rates. Both factors thus contribute to an overlap of the confidence intervals of the estimated growth rates with the other types of aortic root shapes and thus non-significant statistical differences.

Even though aortic shape was not associated with rapid aortic growth in our population, we found that referral for aortic surgery was associated with generalized aortic root dilatation. This finding is in accordance with previous studies: one study revealed that 20/21 patients with generalized aortic root shape suffered from subsequent aortic complications during follow-up [7]. Another recent study described an association of aortic root shape with referral for aortic root surgery in a larger echocardiographic study of 602 children and young adults with Marfan syndrome [15]. This association has clinical implications because generalized aortic root dilatation could be a useful imaging marker for patients at risk of aortic events. Therefore, we believe that a closer surveillance in this subgroup of Marfan patients is needed. However, a change of practice would also need further validation in prospective studies regarding the occurrence of aortic events and/or earlier decision for elective surgery.

Also, it could be interesting to visualize aortic hemodynamics, e.g., by applying four-dimensional flow cardiovascular magnetic resonance imaging. Analysis of abnormal hemodynamic flow patterns and wall shear stress in different shapes of the aortic root could contribute to a better understanding of the underlying pathophysiology in this disease [17, 37]. Previous studies in Marfan patients indicate a potential role of pulse wave velocity and aortic distensibility as early markers of aortic involvement [38–41]. While wall shear stress occurs as a frictional tangential force of flowing blood on the endothelium, wall stress occurs within the aortic wall [42, 43]. Thus, wall stress cannot be derived by imaging measures alone but is dependent of the concurrent blood pressure estimate [42].

Furthermore, it is important to consider that more information than just the aortic dimensions or annual growth rate are needed for the surgeon’s decision to perform an elective surgery. It still is a multifactorial decision and a matter of surgeon´s judgement. Guidelines provide important evidence-based guidance when to operate but the decision making for elective surgery remains complex and may even slightly vary between various centers and surgeons.

Lastly, Z-scores were calculated based on two different populations as provided by Kaiser et al. [29] and Devereux et al. [30], which could compromise its comparability. We chose to use echocardiographic data as provided by Devereux because to best of our knowledge there is currently no MR angiography specific normative data for adult patients. Z-scores based on the methods of Devereux et al. can be applied to patients aged ≥ 15 years. Hence there is an overlap with the age group described by Kaiser et al. possibly resulting in different Z-scores due to the differences in the imaging method and inclusion of patients. In order to minimize this bias, we did not use both methods in the same patient. Also, possible misleading interpretation of Z-scores in children with Marfan syndrome has been discussed in a recent study by Elkinany et al. [44]. As the Z-score is naturally changing even in the absence of any aneurysmal pathology or medical intervention it may decrease spontaneously even in untreated Marfan patients. However, this does not necessarily affect our results as we did not investigate a decreasing Z-score as an effect of a certain treatment.

In summary, larger baseline aortic root diameter and Z-score as well as young age predict progressive aortic root dilatation in Marfan patients. MR angiography derived type of aortic root shape does not predict aortic growth in any segment of the aorta, but patients with generalized aortic dilatation are referred more frequently for aortic surgery.

## Abbreviations

*FBN1*: **Fibrillin-1 encoding gene**
MR: **magnetic resonance**
